# Arthroscopic Arthrolysis, a Minimally Invasive Approach to Treat Arthrofibrosis of the Knee

**DOI:** 10.1016/j.eats.2025.103446

**Published:** 2025-01-31

**Authors:** Julian Kylies, Hendrik Fahlbusch, Elias Brauneck, Dominik Bannier, Markus T. Berninger, Jannik Frings, Karl-Heinz Frosch, Matthias Krause

**Affiliations:** a Department of Trauma and Orthopaedic Surgery, University Medical Center Hamburg-Eppendorf, Hamburg, Germany; b Department of Trauma Surgery, Orthopaedics and Sportstraumatology, BG Klinikum Hamburg, Hamburg, Germany

## Abstract

Arthroscopic arthrolysis is a minimally invasive approach for treating arthrofibrosis of the knee. Arthrofibrosis is a common complication following knee surgery or trauma, resulting in restricted joint motion. Managing arthrofibrosis is challenging and requires various strategies to reduce pain and inflammation while improving range of motion. When conservative treatments fail, more invasive options such as arthroscopic arthrolysis or open arthrolysis may be required. The presented arthroscopic technique successfully restores knee extension, flexion, and patellar mobility while minimizing additional trauma and prevents damage to peripheral structures without causing instability to the knee joint.

Arthrofibrosis is a significant and common complication following knee surgery, resulting in limited joint motion. It affects patients variably following ligament surgery (4% to 13%), fractures in proximity to the knee joint (14% to 50%), or endoprosthetic procedures (5% to 20%).^1^ To date, there is no standardized definition of the term *arthrofibrosis*. However, a commonly used definition is a severe loss of range of motion (ROM) more than 3 months after intervention, an absence of mechanical blockage or infection, and insufficient improvement of ROM by aggressive and painful physical therapy.^1^

The management of arthrofibrosis is complex and involves several strategies for prevention and postoperative care. These strategies aim to reduce postoperative pain and inflammation and restore ROM.^1^ When nonoperative treatment regimen fails, more invasive options like manipulation under anesthesia, arthroscopic arthrolysis, or open arthrolysis may be required.^2^

Although open arthrolysis provides extensive anterior and posterior joint access, it may be less effective releasing focal adhesions close to the articular surface and can lead to excessive scarring again due to the open approach.

The minimally invasive approach of arthroscopic arthrolysis reduces procedural trauma to the knee, resulting in faster recovery, reduced postoperative pain, and satisfactory restoration of knee function.^3^ Recent studies have also shown that it provides superior ROM gains, shorter operative times, and higher patient satisfaction compared to open arthrolysis.^3^ It has already been shown that arthroscopic arthrolysis is effective in successfully gaining back range of the knee joint.^4^ Postoperative timing of the procedure is crucial. The operative strategy and technique is presented in this article.

## Surgical Technique

A video presenting this technique is provided (Video 1). The aim of arthroscopic arthrolysis is to restore a sufficient amount of knee extension, flexion, and patellar mobility in a minimally invasive (arthroscopic) fashion without causing significant additional trauma and instability to the knee joint.

### Patient Positioning and Anesthesia

**Fig 1:**
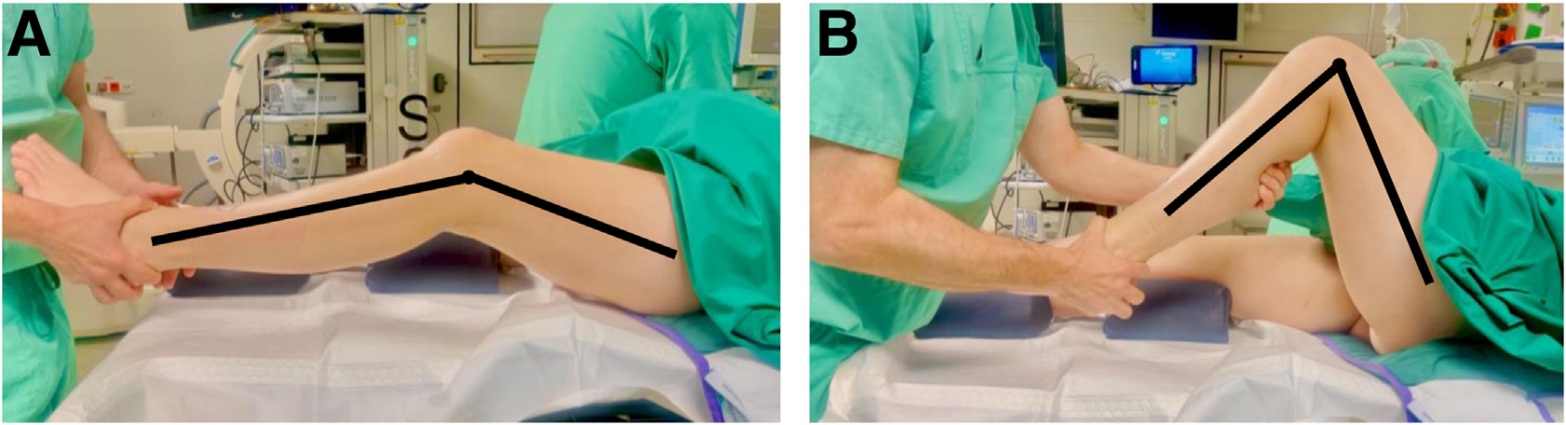
Before arthroscopic arthrolysis, the range of motion and stability of the knee joint are tested. Five months after undergoing revision anterior cruciate ligament reconstruction, a 40-year-old patient exhibits a preoperative range of motion (extension/flexion) of 0°/24°/90° of the left knee.

The patient is positioned supine on the operating table with the affected leg secured in an electric leg holder. We typically also apply a tourniquet but try to limit its use as the quadriceps tension prevents flexion. Options for anesthesia include general, spinal, or epidural. A clinical examination is performed under anesthesia to rule out any instability and to objectify the actual ROM (Fig 1). Pain is controlled during and after surgery with a femoral catheter and/or sciatic nerve block.

### Portal Placement

After patient positioning and sterile draping, anterolateral (AL) and anteromedial (AM) arthroscopic portals are created. In cases of an extension deficit, the arthroscope may not be able to be placed into the suprapatellar recesses. Hence, the AM portal has to be placed first in order to “clean” the femoral notch (including the anterior compartment). Further portals may be necessary during the procedure to clean the posteromedial and posterolateral recesses and perform dorsal capsulotomy. Therefore, in addition to the AL/AM portals, medial and lateral suprapatellar portals, as well as posteromedial and trans-septal posterolateral portals, are added as needed to address scar tissue in all 6 recesses (medial, lateral, posteromedial, posterolateral, suprapatellar, and intercondylar) of the knee joint.

### Diagnostic Arthroscopy and Fibrous Tissue Removal

**Fig 2:**
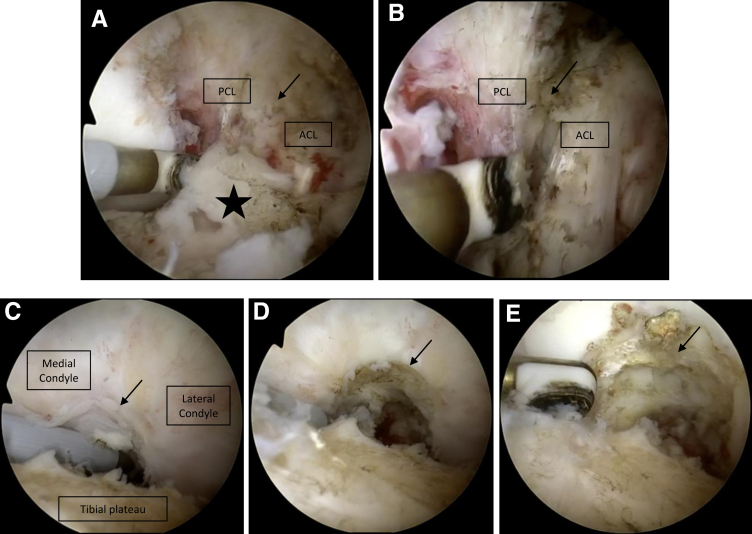
(A) Arthroscopic intra-articular image of the left knee, viewed through the standard anterolateral arthroscopy portal, visualizing parts of the anterior cruciate ligament (ACL) and posterior cruciate ligament (PCL). A radiofrequency electrode was brought in via the standard anteromedial arthroscopy portal. Extensive fibrous tissue formation in the intercondylar notch connects the ACL and PCL, thereby severely restricting range of motion (fibrous tissue marked by a star and arrow). (B) The same arthroscopic view is shown after debriding the fibrous tissue, thereby freeing the ACL and PCL from adhesions (fibrous tissue is marked by an arrow). Caution should be used to avoid damaging these ligamentary structures. (C-E) Arthroscopic intra-articular image of the left knee, viewed through the standard anterolateral arthroscopy portal. A radiofrequency electrode was inserted via the standard anteromedial arthroscopy portal. (C) The femoral notch is visualized before debridement and clearly shows fibrous tissue that occupies both femoral condyles and the tibial plateau (marked by an arrow). Thin layers of fibrous tissue, resembling spider webs, a characteristic scar tissue formation found in knee arthrofibrosis, can be seen (indicated by arrows). (D, E) The same arthroscopic field of view is shown after gradually debriding the fibrous tissue. Excision should continue until fat tissue becomes visible (indicated by arrows).

The procedure is performed with a standard 30° arthroscope (30° 2.7 × 72 mm; Arthrex), an arthroscopic shaver (Excalibur, 4.0 × 13.0 cm; Arthrex), and a radiofrequency electrode (ApolloRF MP90; Arthrex). The initial assessment of the knee joint during arthroscopic arthrolysis involves visualization and mobilization of the anterior cruciate ligament (ACL), the transverse ligament, and the posterior cruciate ligament (PCL) within the intercondylar fossa as they tend to be fused by scar tissue (Fig 2 A and B). Stepwise dissection of the anterior recesses to visualize the ACL and PCL is typically the first challenge. This is carried out using the radiofrequency electrode, carefully beginning at the tibial attachment area of the ACL and separating the transverse ligament. To enhance visualization of the femoral insertions of the ACL and PCL, adhesions between the infrapatellar fat pad and the ligamentum mucosum are debrided. Subsequently, the interval between the ACL and the lateral femoral condyle is developed using an arthroscopic shaver. This preparation is essential for instrumentation and viewing within the posterolateral joint space. Similarly, with the arthroscope positioned in the AM portal and the shaver in the AL portal, the interval between the PCL and the medial femoral condyle is developed. The medial and lateral compartments are inspected for joint and meniscus integrity. Any scar tissue on the cartilage should be gently removed to preserve the articular surface (Fig 2C-E). A common feature of arthrofibrosis is thin layers of fibrous membranes found throughout the knee, covering both the cartilage and the bone (“spider webs”; Fig 2C, marked by arrows). If necessary, the medial, lateral, and superior recesses are exposed, and any scar strands and adhesions are removed. Intermittently, intraoperative ROM assessments are used to check for persisting ROM deficits.

### Dorsal Recess Arthrolysis

**Fig 3:**
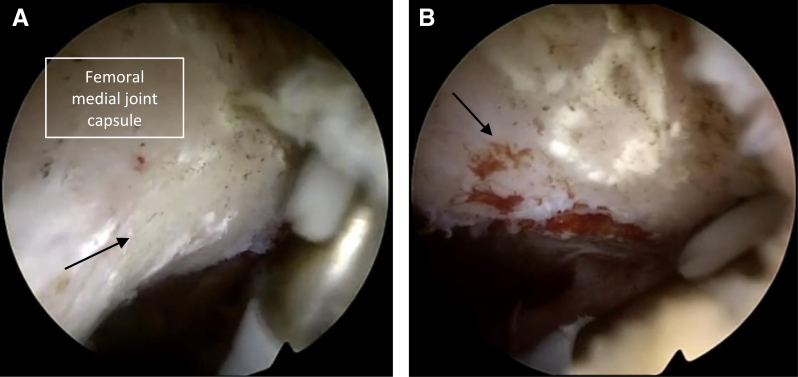
Arthroscopic intra-articular image of the left knee, viewed through the posterolateral portal, pointing toward the radiofrequency electrode brought in from posteromedial. In cases of persisting extension deficits, dorsal capsulotomy should be formed. (A) Dorsal joint capsule attached to the medial femoral condyle (marked by an arrow). (B) Dorsal joint capsule has been removed from the medial femoral condyle (arrow marks bony structures, freed from dorsal joint capsule).

**Fig 4:**
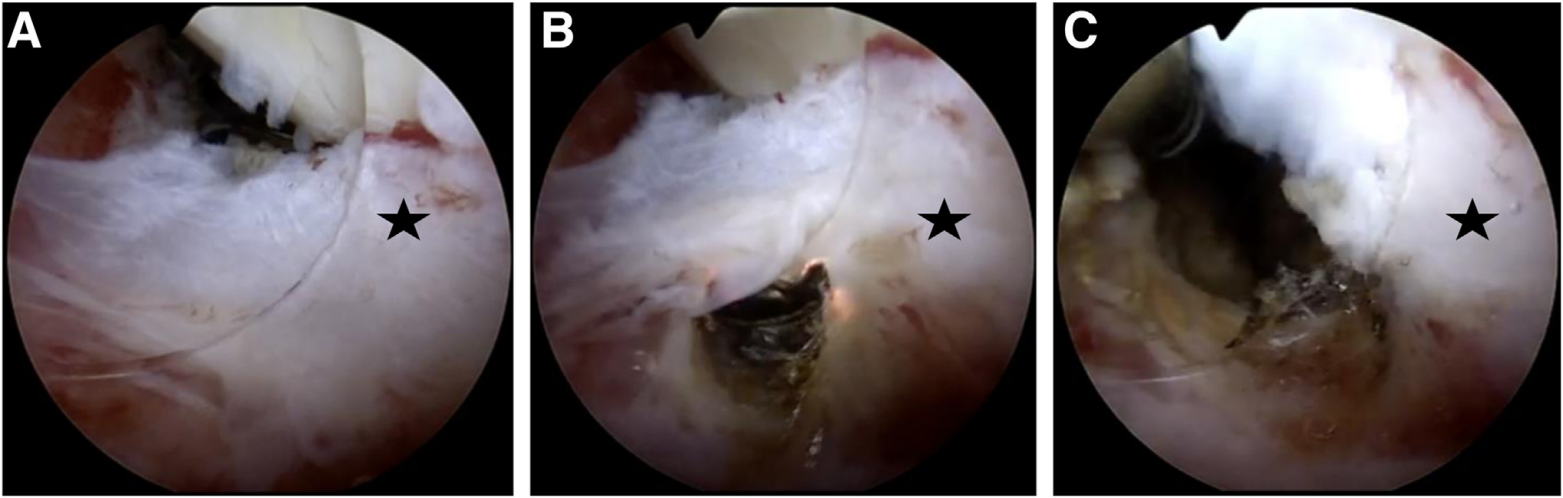
(A) Arthroscopic intra-articular image of the left knee, viewed through the posteromedial portal pointing toward the radiofrequency electrode brought in from posterolateral through a trans-septal approach. Fibrous tissue strands (marked by stars) significantly obstruct the view and the access to deeper areas of the knee. To enhance knee extension and clear the posterior parts of the knee joint, these fibrous tissue strands need to be removed. (B, C) The same arthroscopic field of view is shown after gradually removing the fibrous tissue, thereby allowing the arthroscope to access deeper areas. Care should be taken to avoid vascular injuries in the back of the knee.

If full extension cannot be achieved, posteromedial, posterolateral, and trans-septal portals may be required to release the dorsal joint capsule from the femoral and/or tibial condyles (Fig 3). Posteromedial scar tissue is gradually resected with the goal to rapidly expose the posterior septum. The trans-septal approach can be prepared via visualization between the ACL and PCL or by driving the arthroscope from the AL portal below the released ACL into the posterolateral recesses.^5^ Using the radiofrequency electrode, the trans-septal approach is developed. Switching the arthroscopic view through the posteromedial and trans-septal portal into the posterolateral recesses, the PL portal is placed behind the fibular collateral ligament above the biceps femoris tendon.^5^ Using the radiofrequency electrode, the posterior scar tissue and joint capsule can be removed from the femur, starting right at the cartilage bone junction in the proximal direction (Fig 4). If the anterior recesses and both dorsal recesses are released, including dorsal capsulotomy, full extension should be possible. If not, medial and lateral recesses should be checked, and especially adhesions of the iliotibial band can prevent full extension and flexion as well.

**Fig 5:**
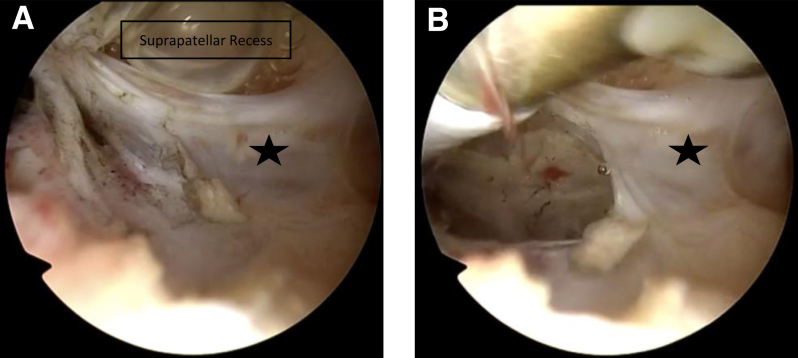
Arthroscopic intra-articular image of the left knee, viewed through the anterolateral portal. The radiofrequency electrode was introduced via the standard anteromedial portal. The suprapatellar region is visualized and a superior capsular release is performed. The suprapatellar region before debridement shows significant adhesions between the quadriceps tendon and suprapatellar fat pad (“white wall,” marked by a star) (A). Using the radiofrequency electrode, the curtain-like adhesions are gradually excised (B) (fibrous tissue is marked by a star).

Once finished in all anterior and posterior recesses, typically the arthroscope can be placed in the suprapatellar recesses in full extension. Typically, a curtain-like scar tissue structure (“white wall”) can be found right behind the entrance of the femoral trochlea. Treatment involves creating initial space with small scissors and gradually enlarging it with a radiofrequency electrode (preferred over the shaver by the authors) to fully excise the scar tissue (Fig 5). If the suprapatellar recesses are completely released, significant improvement in flexion should occur. Depending on the timing of the arthroscopic arthrolysis, between 3 and 6 months postoperatively in most cases, ROM between full extension and 120° of flexion can be achieved intraoperatively.

If, despite extensive arthroscopic arthrolysis, the ROM is still retracted, additional aspects that require open arthrolysis may be considered. If the patella tendon is retracted due to scar tissue formation, likely leading to patella baja and flexion deficit, a proximalization of the tibial tubercle may additionally be performed. Also, a medial release of the patella, where the medial retinaculum is incised using a scissor, may be required to further increase its mobility. However, once opened, critical joint regions, especially the posterior recesses, may not be reachable anymore, so a stepwise arthrolysis is recommended. In severe cases of arthrofibrosis after ACL reconstruction, resection of the graft may also be necessary. If plates are still in situ after osteotomies or fractures around the knee, the plates should be removed if possible (bone healing should have occurred) because they can cause limitation of ROM by themselves and also can induce development of scar tissue (Table 1).

**Table 1:** Pearls and Pitfalls

Pearls	Pitfalls
Place portals carefully in the anteromedial/anterolateral soft spots to avoid complicating scar tissue removal.	Incomplete scar tissue removal might lead to recurrence of ROM deficits.
Consider posterior capsulotomy if extension deficit persists.	Avoid damaging the ACL, PCL, and cartilage when clearing adhesions with ra adiofrequency electrode.
Check for patella tendon retraction and patella baja presence if flexion deficit persists.	Limited access to posterior knee structures may require additional open interventions.
An individual, multidisciplinary postsurgical care approach should be used to ensure the optimal treatment outcome.	Patient compliance is crucial to maintaining postoperative ROM. Patients who cannot participate in the recommended postoperative treatment program may not maintain the ROM gains after surgery.

**Risks**	

Iatrogenic cartilage damage: Due to fibrous tissue complicating arthroscope movement.	
Neurovascular injuries: Especially prone to damage are the saphenous and peroneal nerve as well as the great saphenous vein and popliteal artery.	
Graft damage: Since the ACL and PCL are often fused by scar tissue, care should be taken to avoid damaging these structures while debriding the scar tissue.	
Recurrence of joint stiffness: Despite scar tissue removal, some patients may experience a recurrence of joint stiffness. Postsurgical care plays a vital role in this context.	

ACL, anterior cruciate ligament; PCL, posterior cruciate ligament; ROM, range of motion.

### Postsurgical Care

**Fig 6:**
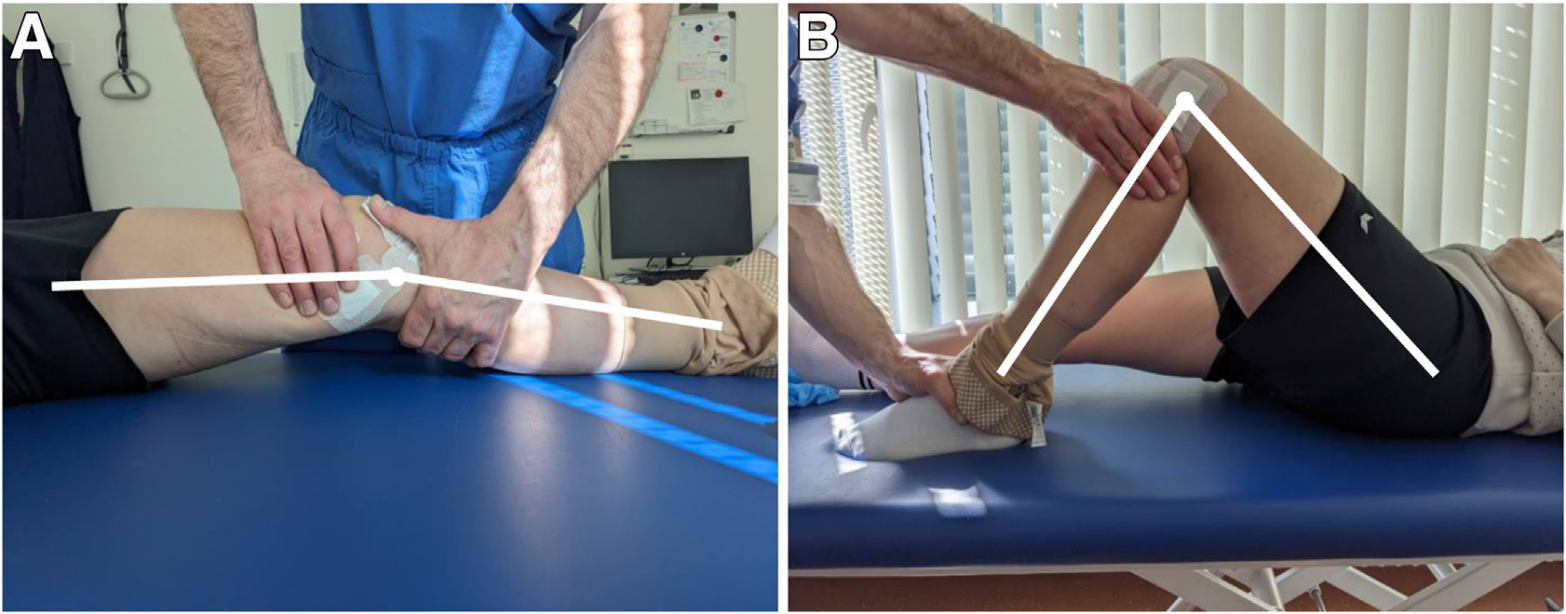
At postoperative day 5 after arthroscopic arthrolysis, the range of motion has greatly improved. The same 40-year-old patient now exhibits a postoperative range of motion (extension/flexion) of 0°/5°/105° of the left knee.

Postsurgical care is complex and should be adapted individually. A multidisciplinary approach involves adequate pain management and gentle, painless physical therapy, including continuous passive motion devices, starting immediately after surgery. Physical therapy plays a vital role in preserving postoperative ROM improvements and should strike a balance between overexertion, which may increase inflammation, and insufficient effort, which could lead to restricted ROM once again. Oral nonsteroidal anti-inflammatory drugs and corticosteroids can be prescribed to minimize the risk of recurrent scar tissue formation.^6^ Figure 6 illustrates the postoperative ROM gain on postoperative day 5.

## Discussion

Postoperative loss of ROM poses a significant challenge following knee surgery. Effective management is vital since limited knee mobility correlates with poor patient outcomes and knee function. In particular, research indicates that inadequate postoperative mobility can lead to long-term issues, with most patients with arthrofibrosis and restricted ROM experiencing severe consequences such as osteoarthritis development of the knee joint.^7^

Following ACL reconstruction, arthroscopic arthrolysis has shown enhanced knee ROM gains by an average of 25°. Moreover, knee function has been shown to improve after arthroscopic arthrolysis as well, as evidenced by enhanced Tegner and International Knee Documentation Committee scores.^8^

The timing of arthrolysis is critical. Prompt arthrolysis within 6 months following the arthrofibrosis-related surgery significantly improves ROM and functional outcomes when compared with delayed arthrolysis.^6^ When comparing minimally invasive arthroscopic arthrolysis with open arthrolysis, it becomes evident that arthroscopic arthrolysis yields superior postoperative ROM gains, faster operation times, less blood loss, and improved patient-reported outcomes.^3^ This might be due to reduced trauma from smaller incisions, allowing immediate knee joint mobilization and minimal risk of wound-healing complications. In addition, the method has shown significant efficacy in addressing infrapatellar contractures and adhesions within specific knee regions, such as the upper medial and lateral recesses and the intercondylar notch.^5^

Nevertheless, the technique has limitations. By using this arthroscopic approach, it is only possible to address intra-articular scar tissue. Adhesions in the periarticular soft tissues cannot be reached through arthroscopic arthrolysis. Moreover, for adhesions in the posterior recess, an arthroscopic approach may not always be sufficient, sometimes necessitating a mini-arthrotomy for complete resolution.

Therefore, arthroscopic arthrolysis is a valuable option for treating knee arthrofibrosis. The procedure shows the best results, when applied within 6 months postsurgery, and can be used synergistically with manipulation under anesthesia. If certain scar tissue compartments cannot be addressed arthroscopically, however, an open approach should be used.

## Disclosures

The authors declare the following financial interests/personal relationships which may be considered as potential competing interests: J.F. has received speaking and lecture fees from CONMED. K-H.F. has received speaking and lecture fees from Arthrex. M.K. has received speaking and lecture fees from Arthrex. All other authors (J.K., H.F., E.B., D.B., M.T.B.) declare that they have no known competing financial interests or personal relationships that could have appeared to influence the work reported in this paper.

## Supplementary Data

Video 1 Before beginning the arthroscopy, an examination of the knee under anesthesia is performed. As presented here, patients have a significant deficit in knee range of motion of the left side. Following proper positioning, sterilization, and draping, an anterolateral and anteromedial arthroscopy portal is created on the suspended knee. The femoral notch is subsequently entered and visualized. A characteristic finding seen in arthrofibrotic knees is the presence of fibrous membranes resembling spider web fibers covering the bone as well as cartilage. Using the radiofrequency electrode, the femoral notch is cleared, allowing for the visualization of cartilage once more. This technique is also applied to clear the medial and lateral femorotibial compartments. Next, the medial and lateral recesses are accessed, and scar tissue is removed. It is essential to stop excising fibrous tissue once fat is visible to prevent triggering the formation of recurrent fibrous tissue. Following this, the anterior cruciate ligament (ACL) and posterior cruciate ligament (PCL) are examined. Here, we observe that the ACL and PCL are connected by fibrous tissue and cannot be distinguished at the moment. This significantly limits knee range of motion. Scar removal must be done precisely to avoid damaging the ACL and PCL, especially from the heat generated by the radiofrequency electrode. To access the posterior aspects of the knee, the posteromedial portal is prepared and established using a twist-in cannula. Subsequently, the posterolateral portal is established under visual guidance. Fibrous tissue is excised here as well. In cases of persisting extension deficits during arthroscopic arthrolysis and extensive posterior fibrosis, a dorsal recess arthrolysis is performed. The dorsal joint capsule is removed from the femur with a radiofrequency electrode starting at the back edge of the condyles and moving from distal to proximal. The dorsal recess is cleared. To assess the suprapatellar recess, medial and lateral suprapatellar portals are created, and the fibrous membranes are cleared. A common finding is the so-called “white wall,” a curtain-like scar tissue structure, which is cleared as well. After performing the arthroscopic lysis of adhesions, the knee range of motion as well as stability is tested once more. Both flexion and extension have greatly improved while still maintaining adequate stability. Arthroscopic lysis of adhesions is a minimally invasive procedure that effectively removes scar tissue within the knee joint, significantly improving range of motion for patients with knee joint stiffness. Postoperative rehabilitation, including immediate gentle physical therapy, is crucial to maintain these improvements and prevent recurrence of adhesions.
